# Development of an Optical System for Strain Drop Measurement of Osteosarcoma Cells on Substrates with Different Stiffness

**DOI:** 10.3390/s24113383

**Published:** 2024-05-24

**Authors:** Ludovica Apa, Maria Vittoria Martire, Serena Carraro, Marianna Cosentino, Zaccaria Del Prete, Barbara Peruzzi, Emanuele Rizzuto

**Affiliations:** 1 Department of Mechanical and Aerospace Engineering, Sapienza University of Rome, 00184 Rome, Italy; ludovica.apa@uniroma1.it (L.A.); mariavittoria.martire@uniroma1.it (M.V.M.); serena.carraro@uniroma1.it (S.C.); zaccaria.delprete@uniroma1.it (Z.D.P.); 2 DAHFMO-Unit of Histology and Medical Embryology, Sapienza University of Rome, 00161 Rome, Italy; marianna.cosentino@uniroma1.it; 3 Bone Physiopathology Research Unit, Bambino Gesù Children’s Hospital, IRCCS, 00146 Rome, Italy; barbara.peruzzi@opbg.net

**Keywords:** *strain drop*, biomechanics, osteosarcoma, strain measurements, stiffness, cell elasticity, novel measurement system, silicone stretchable chambers

## Abstract

Adherent cells perceive mechanical feedback from the underlying matrix and convert it into biochemical signals through a process known as mechanotransduction. The response to changes in the microenvironment relies on the cell’s mechanical properties, including elasticity, which was recently identified as a biomarker for various diseases. Here, we propose the design, development, and characterization of a new system for the measurement of adherent cells’ *strain drop*, a parameter correlated with cells’ elasticity. To consider the interplay between adherent cells and the host extracellular matrix, cell stretching was combined with adhesion on substrates with different stiffnesses. The technique is based on the linear stretching of silicone chambers, high-speed image acquisition, and feedback for image centering. The system was characterized in terms of the strain homogeneity, impact of collagen coating, centering capability, and sensitivity. Subsequently, it was employed to measure the *strain drop* of two osteosarcoma cell lines, low-aggressive osteoblast-like SaOS-2 and high-aggressive 143B, cultured on two different substrates to recall the stiffness of the bone and lung extracellular matrices. Results demonstrated good substrate homogeneity, a negligible effect of the collagen coating, and an accurate image centering. Finally, the experimental results showed an average *strain drop* that was lower in the 143B cells in comparison with the SaOS-2 cells in all the tested conditions.

## 1. Introduction

In recent years, the mechanical properties of cells, particularly the role of cell elasticity, have captured the attention of numerous experts, including biologists, engineers, and clinicians. Cell stiffness refers to the capacity of a cell to resist applied deformations [1], and cell elasticity allows us to gain insights into various pathological conditions [2]. Indeed, cells are significantly influenced by their surrounding microenvironment, adapt to and respond to physical and mechanical stimuli to maintain homeostasis [3,4], and can perceive mechanical cues to convert them into biological responses with an intracellular process known as mechanotransduction [5]. Interestingly, their reaction varies according to the cell type and, in particular, to their intrinsic stiffness [6]. Generally speaking, cancer cells are known to induce a stiffening of the tissue in which they develop, and cancer-associated fibrosis is a critical component of the tumor microenvironment which significantly impacts cancer and normal cell behavior [7]. Nonetheless, literature studies propose contradictory experimental results, reporting lower [8], unchanged, or increased stiffness in pathological cells [9]. These contradictory results are also dependent on the type of cancer, given that the range of elasticity changes among different tumors, the malignancy degree, and according to the stiffness of the matrix with which they interact [10,11]. Therefore, the interaction between adherent cells and their surrounding substrate plays a crucial role, in particular for those diseases based on changes in cell mechanics [12].

As for cell stiffness, several techniques are available for its measurement, such as the Optical Stretcher, Micropipette Aspiration, and Atomic Force Microscopy, and each of them has *pros* and *cons*, such as their invasiveness [13], being time-consuming, and the need for specialized equipment [14]. Interestingly, Bartalena et al. [15] have developed a new non-invasive method for measuring a stiffness-related parameter of individual adherent cells, the *strain drop* [16], determined as the deformation of the substrate under each single cell when subjected to an external stretch. The *strain drop* is a parameter directly related to cell elasticity and takes into account the capability of the cell to adhere to the substrate and resist its deformation. Therefore, it also includes the biophysical aspects of cellular processes, such as intracellular signaling, actin cytoskeleton activity, and changes in cell volume and morphology [17,18]. In view of this, even if the *strain drop* does not provide a direct measurement of cell elasticity, as with Young’s modulus or the storage modulus, it is highly suitable for studying adherent cells. Similarly, Fu et al. [19] estimated the nominal spring stiffness of the PDMS microposts underneath the cells to study the impact of their rigidity to cells’ morphology and adhesion.

Within this context, here we developed a new simple and accurate technique to measure the *strain drop* of adherent cells seeded on substrates with different elasticities, thereby implementing the cell–matrix interplay on the cells’ stretching. The technique is based on the linear stretching of a silicone culture chamber ad hoc modified to recreate microenvironments with different stiffness values, a feedback system for image centering, and a high-speed camera mounted on an inverted microscope for image acquisition. The technique was then employed to study two osteosarcoma (OS) cell lines with different aggressiveness [20]. OS is an aggressive bone tumor that originates from primitive bone-forming cells known as osteoblasts [21]. OS predominantly affects children and young adults and develops mainly in the long bones like the tibia, humerus, and femur [22]. About one out of five osteosarcomas are metastatic at diagnosis, putting the 5-year survival rate below 30%, due to the spreading to other tissues in the body, mainly the lung parenchyma [23,24], a tissue with a mechanoenvironment (e.g., stiffness) significantly different from that of the tissue of origin. In this context, the interplay between cells and the tissue-specific extracellular matrix (ECM) plays a crucial role in unraveling the mechanisms behind the metastasis development. The ECM is a meshwork of macromolecules and minerals that surrounds, supports, and gives structure to cells. Of note, cancer development is known to alter the ECM of the involved tissues/organs, thereby inducing a modulation of the mechanoenvironment [25]. In vitro studies revealed that cells sense the intrinsic mechanical properties of the substrate on which they adhere and respond accordingly by modulating cell homeostatic processes [26,27,28]. Deregulation in the expression of cell mechanosensor proteins or in the tissue mechanoenvironment correlates with pathological conditions [11,29,30], further highlighting the fine-tuning regulated interplay between the cell and ECM stiffness. At this point, it has to be noted that cell substrate deformation is a crucial mechanical stimulus experienced by cells in vivo [31], in particular for bone cells, neurons, and epithelial, skin, and muscle cells. Finally, it has to be remarked that the cell–matrix interplay is not only related to substrate stiffness, but also to the intrinsic cell elasticity machinery. Indeed, since our aim was to characterize the elasticity of the two selected OS cell lines on substrates with different stiffnesses, we chose to focalize the measurement of the *strain drop*, which also allowed us to take into account the cell–matrix interplay. The cell–matrix interplay was shown to be crucial in mediating cell signaling, proliferation, differentiation, migration, and cell fate [25,32,33,34,35]. Of note, the modulation of cell elasticity from a physiological tissue-specific condition has been recently proposed as a marker of diseases for various pathologies [9], such as oncological transformation, Alzheimer’s disease, and vascular diseases.

The subsequent sections are structured as follows: Section 2 provides an overview of the design, development, and characterization of the testing system and of the experimental process, including the procedure and the statistical analysis. Section 3 and Section 4 present and discuss the obtained results, respectively, while Section 5 summarizes the conclusions and significant achievements.

## 2. Materials and Methods

### 2.1. In Vitro Stretching Device

**Figure 1:**
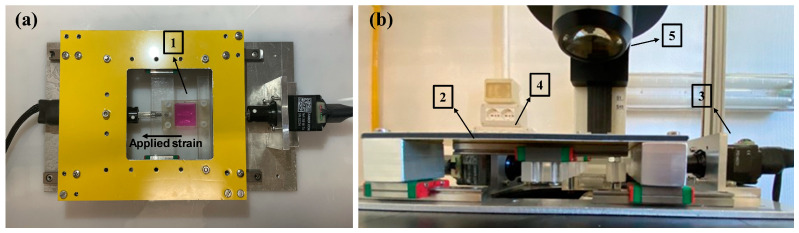
Experimental set-up for cell elasticity measurement. (**a**) Detail of the stretching system: 1 Stretch chamber; (**b**) Experimental set-up: 2 Main linear actuator; 3. Second actuator; 4. The stage composed of the main actuator and the stretch chamber; 5. Inverted microscope with a high-resolution camera.

The in vitro stretching system was designed to measure the cell’s *strain drop* by a non-invasive optical method during the stretching of *custom-made* silicone chambers, whose fabrication is detailed in Section 2.2. The device consists of two linear actuators (NA11B16-T4, Zaber, Vancouver, British Columbia, Canada), both connected to a stepper motor controller (X-MCB2, Zaber, Vancouver, British Columbia, Canada) and placed on two linear sliding guides [36]. The first actuator is used to stretch the silicone chamber to obtain the desired deformation of the substrate, while the second one is responsible for returning the image field of view to the initial position by moving the entire system, as shown in Figure 1. The system is completed by a high-resolution camera (acA2040-180 Km, Basler, Ahrensburg, Germany), pixel size: 5.44 μm × 5.44 μm) incorporated into an inverted stereomicroscope (DM IRB LEICA, Wetzlar, Germany) for real-time image acquisition, a personal computer equipped with an NI PCIe1433 frame grabber (National Instruments, Austin, TX, USA), and a NI PCIe 6353 (National Instruments, Austin, TX, USA) data acquisition board. Images were acquired in the widefield through a plan-APO objective at a magnification of 40× and a maximum working distance of 20 mm.

The LabView2018 software was developed to synchronize the movements of the two linear actuators with image acquisition. Specifically, the program was set to apply a nearly static ramp signal with low speed, and a value of amplitude related to the local deformation desired on the chamber’s surface. During cell stretch, an optical tracking algorithm [37,38,39] measured the actual displacement of the individual cell in real-time, thus providing the input for the movement of the second actuator, which is in charge of the repositioning of the system within the image field of view (FOV). The real-time displacement measurement and control system was implemented using the LabVIEW VI “IMAQ Optical Flow”, available in the LabVIEW library. This VI uses the Lucas and Kanade pyramid algorithm to analyze motion in a series of images [40,41] and calculates the positional changes in the reference points (pixels) between consecutive frames, assuming a relatively constant flow in the local pixel neighborhood.

**Figure 2:**
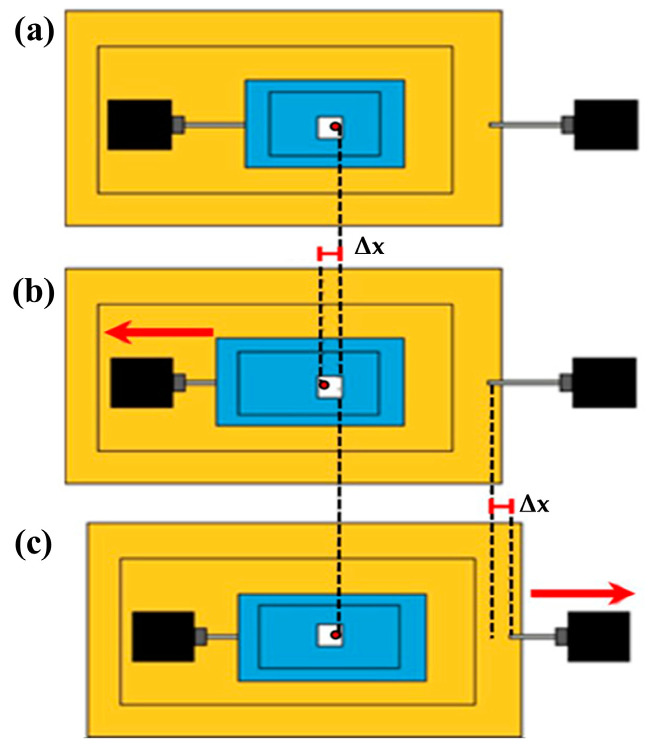
Scheme of the dual-motor stretch system mechanism for cell centering. (**a**) Pre-stretching: the cell is in the image field of view; (**b**) Substrate stretching: motor 1 (left) induces the desired strain on the elastic chamber, with a displacement of Δx of the cell; (**c**) Motor 2 (right) is in charge of the movement of the entire system of the same quantity of Δx in the opposite direction so to have the cell centered in the FOV for image acquisition. The red dot represents a specific pixel on the chamber, which serves as a reference point for the tracking algorithm.

The procedure to center the cell with respect to the FOV is schematized in Figure 2. The silicone chamber (in blue), designed for in vitro cell culture, is secured between the plexiglass plates. When the first linear actuator is activated, the substrate undergoes deformation. A pixel is selected (e.g., the red dot in the figure) to serve as a reference point for the tracking algorithm (Figure 2a). Following the uniaxial elongation to the left, with a displacement of Δx (Figure 2b), the algorithm measures the displacement of the reference pixel, which represents the input for the second motor capable of moving the entire system in the opposite direction (Figure 2c). This procedure ensures that the cell under test remains centered in the image FOV during the stretching process.

### 2.2. Fabrication and Characterization of Chamber Substrate with Different Stiffnesses

**Figure 3:**
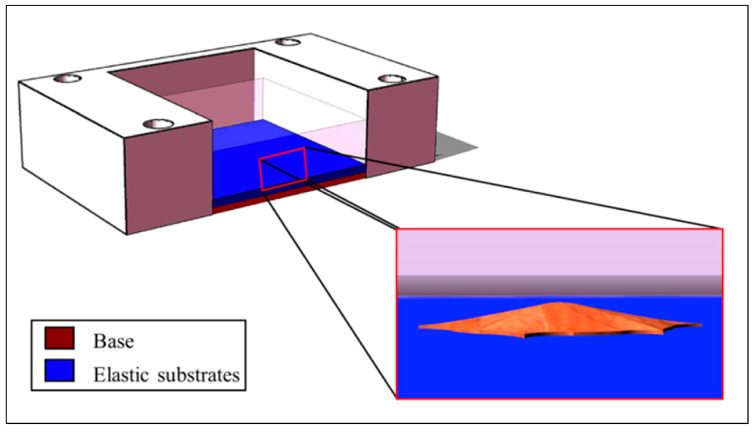
Scheme of the culture stretch chamber, composed of the base and the elastic substrates, with the magnification of an adherent cell.

To unravel the effects of the interplay between the underlying matrix rigidity and the adherent cells, two different testing chambers were realized by manufacturing different layers into the culture stretch chambers. As reported in Figure 3, each chamber was assembled by incorporating a *custom-made* base with a suitable stiffness (300 kPa on average [42]) on a commercial external support (STB-CH-04, Strex Inc., San Diego, CA, USA). The base was formulated by mixing the monomer and cross-linking agent of Sylgard 184 (Dow Corning, Midland, Michigan) in a 10:1 ratio, with a total solution volume of 0.240 mL (to be poured on a surface of 4 cm^2^, with a resulting height of about 600 µm). A thin elastic substrate with a height of about 200 µm was then applied over the base. Given that the OS cells employed in this study, and described in the following Section 2.4, differ in their metastatic potential, we aimed at studying their elasticity in the native environment (bone tissue) and the metastasis-targeted tissue (lung). As a consequence, we developed substrates of 30 kPa and 5 kPa of elasticity, resembling the bone osteoid surface and the lung parenchyma, respectively. For the elastic substrates, to achieve an elasticity of 5 kPa, we employed the bi-component silicone gel Sylgard 527 (Dow Corning, Midland, Michigan) with a ratio 1:1 of the two components. On the other hand, a mass ratio of 1:20 of Sylgard 184:527 was used to obtain an elasticity of 30 kPa. The total volume employed for the elastic layers was 0.080 mL (to be poured on a surface of 4 cm^2^, with a resulting height of about 200 µm). All the prepared mixtures were cast on a specific chamber support on a glass slide, and thermal polymerization was conducted at 125 °C for 75 min.

**Figure 4:**
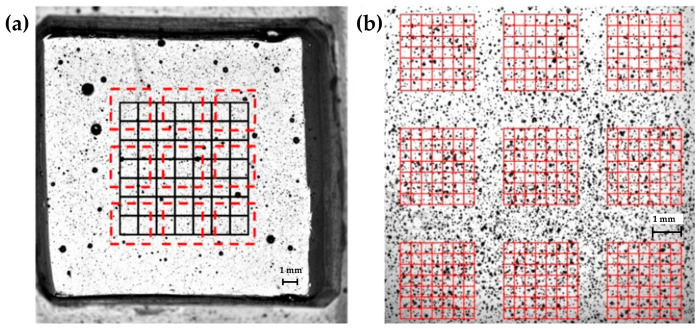
Regions of interest identified for the local and global homogeneity analysis. Stretching chambers were previously treated with a black spray paint to obtain a suitable pattern. (**a**) Black lines indicate the 840 × 840 pixel ROI previously employed for the global homogeneity analysis. Red dashed lines represent the nine ROIs here employed for the local homogeneity analysis. (**b**) Magnification of the nine 490 × 490 pixel ROIs with the highlighted 64 nodes employed for local homogeneity analysis. Figure 4a has a magnification of 0.5× and Figure 4b has a magnification of 1×, acquired through a Nikon SMZ800 microscope (Nikon Corporation, Minato-ku, Tokyo, Japan).

In a previous work [42], a characterization of substrate stiffness perceived by adherent cells was conducted using tensile and indentation techniques. The average values of substrate elasticity were found to be 314.90 ± 31.88 kPa for base samples, and 5.33 ± 0.74 kPa and 29.84 ± 0.99 kPa for the elastic samples, respectively, which are in high agreement with the desired values (5 kPa and 30 kPa). Simultaneously, the substrates were characterized in terms of the global strain homogeneity. Briefly, a region of interest (ROI) of 1600 pixel × 1600 pixel was chosen and divided in 64 nodes for 5 kPa and 30 kPa culture bases, previously treated with a black spray paint to obtain a suitable pattern for Digital Image Correlation analysis, an optical method widely used for strain measurement [43,44,45], as shown in Figure 4a. Chambers were stretched at 1%, 2%, 3%, and 4%, and the images were acquired before and after each elongation for a post-processing analysis of the deformation that occurred in each node. Results showed a variance, expressed in terms of the ratio between the standard deviation and the average strain across the 64 nodes of the center 840 × 840 pixel ROI, which was always lower than 10% for the 5 kPa bases, and always lower than 7% for the 30 kPa bases.

In this manuscript, we proceeded with a local evaluation of the deformation that occurred on the substrate to deeply investigate the surrounding of each single cell. The same uniaxial stretching system [36] was engaged to apply longitudinal strains and the high-resolution camera was employed for real-time image acquisition. At first, the chamber to be tested was aligned with the optical camera. Then, it was stretched to induce different values of local strain; in particular, we measured the local homogeneity at 1% and 4% of stretching. For the entire duration of each test, the images (2040 × 2040 pixels) were acquired at 100 fps with a magnification of 1X and then post-processed through the DIC. Once again, the sample area was previously sprayed to obtain the desired speckled pattern. In detail, the pattern had an average particle radius of 2 ± 1 pixels (22 μm ± 11 μm), and showed a coverage factor, namely, the ratio between the gray pixels over the total image pixels, of 59%. To detect the local strain distribution, we identified 9 ROIs included in the region of interest previously chosen for the global homogeneity, as shown in Figure 4a. Images were acquired with an optical magnification of 1× and each grid had a size of 490 pixel × 490 pixel (2695 μm × 2965 μm) with 7 × 7 subsets (64 nodes), as shown in Figure 2b. Three samples for each type of base were tested.

### 2.3. Validation of Real-Time Algorithm for the Substrate Displacement Measurement

The accuracy of the system in repositioning the image within the field of view, representing the initial position of the cell, was assessed by evaluating the residual error measured by the algorithm after any applied strain. A series of experiments were conducted using four chambers, two for each of the two substrate types. Microparticles of 1.5 μm of diameter were incorporated into the elastic substrate of the chambers, facilitating the displacement measurement for the algorithm validation. On the basis of a series of preliminary tests, we determined the microparticle concentration to obtain a number of items between 20 and 50 on the same layer for each of the nine selected regions, allowing for a good identification of a single microparticle. For each chamber and substrate type, nine equidistant zones were identified in the central part of the chamber, approximately the same ROI employed for the local homogeneity assessment, and for each zone, three single microparticles were selected. The *x* and *y* coordinates of the initial position of each microparticle were set into a LabVIEW program developed ad hoc for the displacement measurement. The assessment of the camera repositioning error within the field of view during the deformation was conducted at each strain step, namely, 1%, 2%, 3%, and 4%. Manual focus was performed at each step, identifying the same initial microparticle. Of note, we previously evaluated that these small focus adjustments induced negligible (<2.5% of the applied strain) changes in the *x-y* localization of the pixels inherent with the measurements. Desired strain and linear actuator velocity were controlled by the LabVIEW program. Images with dimensions of 2040 × 2040 pixels were acquired at 100 fps, with a magnification of 40×. The mean percentage error $\bar{e_{i}}$(%) in camera repositioning within the initial field of view was calculated as the average of the percentage errors ($e_{i}$(%)) for the nine zones at each of the 4 applied strain values, considering three pixels (one for each microparticle) for each zone, for two chambers of each substrate type, for a total of 27 values for each tested condition.

(1) $$e_{i}\left(\%\right)=\frac{P_{x0}-P_{xi}}{P_{x0}}\times 100$$

where $P_{x0}$ is the starting position along the *x*-axis of the chosen pixel at 0% of strain and $P_{xi}$ is the final position along the *x*-axis at *i*% of strain, with *i* = 1, 2, 3, 4.

(2) $$\bar{e_{i}}\left(\%\right)=\frac{1}{n}\;\sum e_{i}\;(\%)$$

where *i* = 1, 2, 3, 4 and *n* are the number of considered points (pixels).

The values obtained from Equation (2) for each substrate elasticity and for each applied strain were then used as correction factors within the LabVIEW2018 control software to optimize the system. After that, to evaluate the goodness of the correction, we tested the other four chambers, two with an elasticity of 5 kPa and two with an elasticity of 30 kPa. For each chamber, we considered three different zones and tested three pixels for each zone (with a total of 9 values for each tested condition) and calculated the average percentage errors as in Equation (2) for the applied strains of 1%, 2%, 3%, and 4%.

### 2.4. Cell Culture Preparation

**Figure 5:**
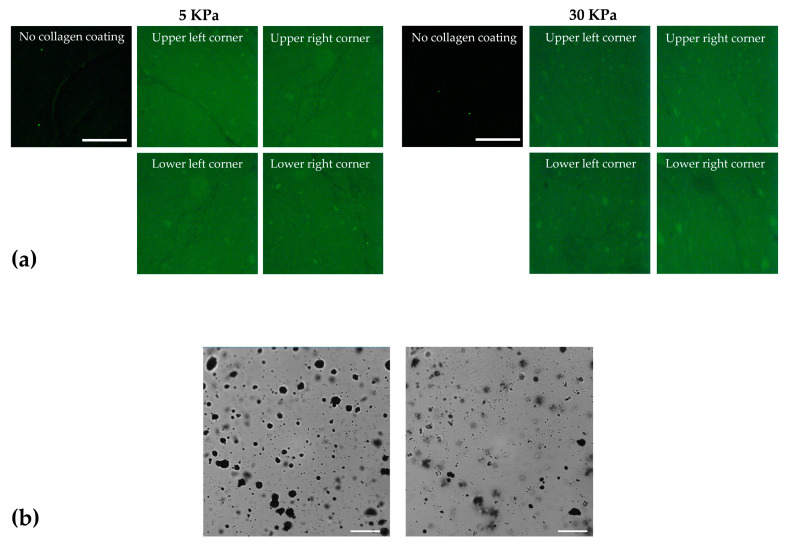
(**a**) Representative images of the immunofluorescence analysis of the collagen coating on 5 kPa and 30 kPa silicone chambers in comparison with non-coated chambers. Images were acquired at 4×—scale bar: 900 µm. (**b**) Example of the images acquired to test the influence of the collagen coating on strain transmission. Left: the focus is on the speckle above the collagen layer; right: the focus is on the speckle below the collagen layer. Images were acquired at 40×—scale bar: 40 µm. Images in (**a**) do not contain any speckle pattern.

Two human pediatric osteosarcoma cell lines with different degrees of malignancy were employed for the stretching experiments. The cell line SaOS-2 (catalog # HTL01001) was purchased from Banca Biologica and Cell Factory (IRCCS Azienda Ospedaliera Universitaria San Martino-IST, Genova, Italy), and the 143B cell line was purchased from American Type Culture Collection (ATCC University Boulevard Manassas, Manassas, VA, USA, 20110). SaOS-2 and 143B are, respectively, low-aggressive osteoblast-like cells [46] and cells with a high malignant potential which usually metastasize to the lung [47]. Cells were grown in Petri dishes with Dulbecco’s modified Eagle’s medium (DMEM, Euroclone, Milan, Italy) supplemented with 10% Fetal Bovine Serum (FBS) and 100 units/mL penicillin/streptomycin (Euroclone, Milan, Italy). Cells were maintained at 37 °C in 5% CO_2_ until the desired confluence. For each experiment, the silicone chambers were pre-stretched (10% of their initial length) with fixed supports and then sterilized by UV exposure for 20 min. After that, the chamber’s substrate was functionalized with a thin layer of the organic protein collagen-I (Sigma Aldrich, St. Louis, MI, USA) to allow for cell attachment. In more detail, 1.5 mL of a 1 mg/mL calf skin collagen solution in 0.1M acetic acid was poured on the substrate’s surface, and the functionalized stretch chambers were incubated at 37 °C in 5% CO_2_ for 3 h. The optimal collagen concentration was determined according to the product datasheet and preliminary experiments. At the end of the coating procedure, the liquid in excess was removed and the remaining solution allowed to evaporate overnight in the laminar flow hood, including 20 min of UV sterilization. Preliminary experiments showed that, at the end of this treatment, the collagen was uniformly distributed over the silicone chambers, as shown in Figure 5a. In detail, to visualize the collagen I coating, substrates were stained following the above protocol, with 1:500 primary mouse monoclonal anti-collagen I-α1 antibody (NB600-450 Novus, Minneapolis, MN, USA) and 1:500 Alexa488 goat anti-mouse secondary antibody (A11017, Life technologies, Carlsbad, CA, USA). As the negative control for the experiments, the substrates were stained with primary and secondary antibodies with no previous collagen coating. Images were acquired by a motorized inverted fluorescence microscope, the DMi8 Leica, with a Hamamatsu Orca Flash4.OLT camera. For the cellular experiments, the cells were plated with a concentration of 8 × 10^3^ cells in each chamber [15] (2000 cells/cm^2^), and the chambers were then maintained at 37 °C in 5% CO_2_. All experiments were conducted 48 h after plating.

### 2.5. Assessment of Induced Strain over Collagen Layer and Technique Validation

Since the collagen coating introduced a thin layer between the silicone substrate and the cells, preliminary experiments were performed to verify that this layer did not alter the strain perceived by the cells with respect to that induced by the substrate. In particular, we chose to test the 5 kPa chambers since, due to their lower stiffness, they are more deformable during stretching, thereby more subjected to any possible alterations in the strain field during the test. One 5 kPa chamber was treated to obtain a speckle pattern directly on the silicone substrate and one over the collagen layer. Using the stretching device, we subjected the chamber to deformations of 1%, 2%, 3%, and 4%. For each step, we captured one image for the speckle pattern below and above the collagen layer. Of note, the two speckles were clearly distinguishable due to the different working focus, as shown in Figure 5b.

Five zones were considered, and for each zone, we measured the displacements of 3 different points, on average, for all the applied strains.

Subsequently, with the aim to perform a validation of the sensitivity of the measurement technique, we measured the *strain drop* of untreated 143B cells in comparison to 143B cells previously treated with glutaraldehyde (GA) (3% in PBS) [15,48,49] to make the cells stiffer than the control cells by creating chemical bonds between the cell and the substrate [50], or with cytochalasin-D (CD) (0.5 μg/mL in DMSO) to inhibit the assembly of actin filaments, reducing the cells’ ability to withstand stress [51] when plated on 5 kPa substrates. GA and CD treatments lasted 20 min and 15 min before the beginning of experimental testing, respectively.

### 2.6. Experimental Protocol for Cell Elasticity Measurement

After the cells were seeded in the *custom-made* silicone chambers, a resting time of 48 h was provided to allow for the proper adhesion of the cells. The chambers to be tested were then mounted in the stretching device. With the aim of measuring the *strain drop*, and to explore the impact of coupling different substrate stiffnesses with matrix deformation on the response of adherent osteosarcoma cells, we subjected the cells to a 1% stretch followed by a 4% one, with a constant rate of 0.1 mm/s. For the entire duration of the tests, images with a resolution of 2040 × 2040 pixels were captured at a magnification of 40×. To optimize the image acquisition and subsequent processing, small manual focus adjustments were performed after each iteration to compensate for the unavoidable changes in focus due to the very high magnification. The experimental protocol proceeded as follows: an image of the cell in rest position was acquired, and then another image was acquired following each iteration at 1% and 4%. To be considered for analysis, the cell needed to exhibit strong adhesion to the silicone base, and the images needed clear contrast and proper focus. Indeed, for each iteration, we considered both the undeformed image (0%) used as a reference and the deformed (1% and 4%) images obtained after the stretching.

### 2.7. Image Analysis and Strain Drop Calculation Protocol

**Figure 6:**
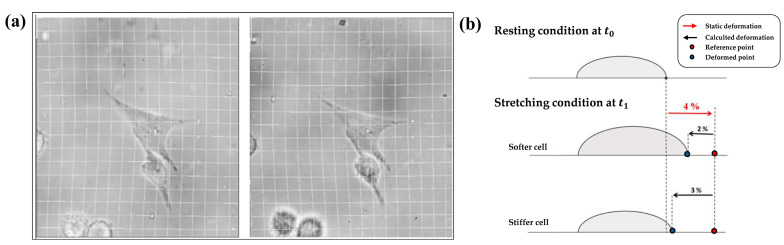
(**a**) Example of an elastic imaging process with the visualization of undeformed (left) and deformed (right) cells at 3% of deformation. (**b**) Example of an experimental test, in which the input was a 4% static deformation on the substrate: resting condition of a generic adherent cell (up) and the stretched condition of the same cell after the application of the external strain in the case of a *soft* (center) and *stiff* (down) cell.

The images acquired during the tests underwent a pre-processing using an image editor software with the sole purpose of ensuring a correct overlap between the reference image A and the deformed ones B, as shown in Figure 6a. Once a correct image superimposition was obtained, we employed software (ImageJ 1.53t bUnwarpJ plug-in [52]) to measure the substrate strain field, which returned the deformation field for each strain imposed during the experimental test. The algorithm allowed for the calculation of the elastic transformation of the deformed image into the undeformed one. Image A was hypothesized to be elastically deformed by a B-spline to appear as similar as possible to image B, working in indirect mode. Through the program dialog box, the following settings were chosen: “Mono” recording mode and “Very Fine” and “Super Fine” settings for the initial and final images, respectively. At the end of the analysis, the software created a “Direct Transformation” file, presented in matrix form, in which each cell represented the X and Y displacements of each *i*-th pixel of the deformed image B in relation to the reference image A. These data were then processed through a MATLAB code ad hoc developed to obtain the entire 2D deformation field, and the color field of the strain was plotted. The *strain drop* value was then finally extracted by locating the cell in the color field, setting the color bar ranges appropriately with reference to the deformation performed, and extrapolating the minimum value of the strain matrix. The *strain drop* is a differential parameter that quantifies the strain that the substrate reaches due to cell presence in comparison to what it would have reached without any cell adherent on it (i.e., the strain imposed by the device). If, for example, we consider a single cell adherent to the substrate under resting conditions (Figure 6b up), the corresponding image acquired represents the undeformed reference one. After applying a 4% static strain to the elastic substrate, the cell responds to the strain by withstanding the applied load, and the substrate underneath the cell deforms less than the other part of the chamber where no cells are seeded. A differential *strain drop* (negative for the way it is computed) can therefore be measured, being higher in absolute value, whereas the cell is stiffer and prevents the underneath substrate from deforming freely. The maximum *strain drop* of the substrate underlying each cell was taken as representative of the entire process [15].

### 2.8. Statistical Analysis

All of the statistical analyses were carried out with GraphPad Prism 8.0.2 and the differences were considered significant when *p* < 0.05. The strain values for the evaluation of the local homogeneity are shown as the mean ± standard deviation (SD) of the 9 ROIs calculated for the two considered stiffnesses and for 1% and 4% of applied strain. The values associated with each single ROI is the average of the values of the 64 nodes. The coefficient of variation (CV) was computed as the ratio of the standard deviation to the mean values. The mean percentage errors values, calculated as Equation (2), are shown as the mean ± standard deviation (SD) of 27 and 9 repetitions for the evaluation of the real-time algorithm accuracy and the subsequent algorithm correction, respectively, calculated for eight combinations of the applied strain and substrate elasticity, and performed on two different silicone chambers. A repeated-measures two-way ANOVA was performed for each silicone chamber assuming the *applied strain* and *substrate elasticity* as *fixed factors*. The displacement values measured on the elastic substrate are shown as the mean ± standard deviation (SD) of at least 9 repetitions calculated for the four applied strains on the elastic substrate and on the elastic substrate with the collagen layer. A two-way ANOVA was performed assuming the *applied strain* and *substrate elasticity* as *fixed factors*. All the maximum *strain drop* values calculated on the SaOS-2 and 143B cell lines are shown as mean ± standard deviation (SD). As for the validation of the sensitivity of the technique, the maximum *strain drop*s were measured for the 143B cell lines cultured on the 5 kPa substrate elasticity (*n* ≥ 6), and an ordinary one-way ANOVA was performed, followed by a multiple comparisons test to look for differences in the cellular mechanical treatments. As for the experiment for the *strain drop* measurements, the maximum *strain drop*s were measured for the 143B and SaOS-2 cell lines cultured on 5 kPa and 30 kPa of substrate elasticity (*n* ≥ 6) and subjected at 1% and 4% of strain. A Mann–Whitney test was performed to look for significant differences in the maximum *strain drop* between the two types of cell lines at each tested condition of applied strain and substrate elasticity. Of note, for a mere graphical improvement, the *strain drop* values have been considered in modulus.

## 3. Results

### 3.1. Local Strain Homogeneity Evaluation

Table 1 shows the mean value, standard deviation (SD), and the coefficient of variation (CV) of the axial strain measured for the three tested samples of the 5 kPa elastic substrate for each stretching condition. Results showed that the average strain value for each tested sample approximated the applied strain with a percentage error always lower than 6%. As for the homogeneity, the coefficient of variation in the strain measured on the nine ROIs was found to be always lower than 11.2% for 1% and 4% of applied strain. These results pointed out a good local strain homogeneity and are in complete agreement with the results previously obtained for the global homogeneity analysis [42], where we found coefficient of variation values always lower than 10%, on average, for all the tested strain conditions.

Table 2 shows the mean value, standard deviation (SD), and the coefficient of variation (CV) of the axial strain measured for the three tested samples of the 30 kPa elastic substrate for 1% and 4% of applied strain. Similarly to the 5 kPa substrates, experimental results showed average axial strain values very close to the desired ones, with a percentage error always lower than 4%. The CV computed on the nine ROIs resulted as lower than 9% in all the tested conditions. Again, these results are in agreement with the measurements for the global analysis, in which the coefficients of variation obtained for the substrate at 30 kPa were always lower than 7%.

### 3.2. Validation of the Substrate Displacement Measurement Algorithm

**Figure 7:**
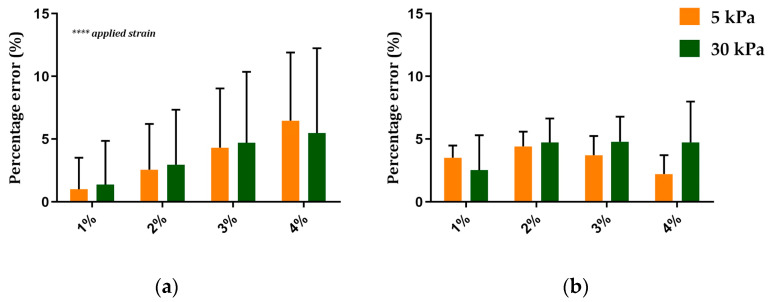
(**a**) Mean ± SD of the percentage error computed on a single silicone chamber obtained for each applied strain and for the two different substrate elasticities, *n* = 27. (**b**) Mean ± SD of the percentage error computed on the same single silicone chamber for each applied strain and for the two different substrate elasticities after the algorithm correction, *n* = 9. ****: *p* < 0.0001.

Figure 7a displays the mean and SD of the percentage error values calculated on a single silicone chamber, as in Equation (2), and computed for the three selected pixels and the nine zones for each applied strain and substrate elasticity.

Results showed that the average error value increased for the strain increases for both of the tested substrates. Specifically, the minimum percentage error was found when stretching the silicone chamber at 1%, with an average value of around 1% and 1.4% for the 5 kPa and 30 kPa substrate elasticities, respectively. The maximum error occurred at 4% of applied strain, with an average value of about 6.5% and 5.5% for the 5 kPa and 30 kPa substrate elasticities. Two-way ANOVA showed a significant increase in the percentage errors when increasing *applied strain* (*p* < 0.0001) but not when varying the *substrate elasticity* for the two tested silicone chambers.

Figure 7b shows the average value and the SD of the percentage errors, calculated as in Equation (2), on the same silicone chamber used for the evaluation of the system accuracy after the algorithm correction. Results showed a reduction in the percentage errors for all the combinations of the applied strain and substrate elasticity, resulting in errors of lower than 4%. Interestingly, after the algorithm correction, the results also showed a reduced standard deviation for all the tested conditions, pointing out an increased repeatability of the results. As expected, the two-way ANOVA showed no significant differences in the percentage errors for the *applied strain* and the *substrate elasticity* for both the tested silicone chambers.

### 3.3. Validation of the Measurement Technique for Cell Strain Drop Measurement

**Figure 8:**
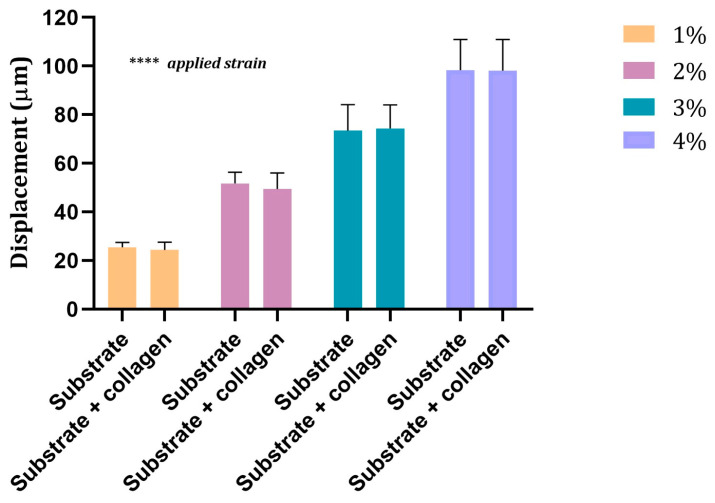
Displacement values measured on the substrate of 5 kPa of elasticity below (substrate) and above (substrate + collagen) the collagen layer for the four applied strains. Values are mean ± SD. 9 ≤ *n* ≤ 14. ****: *p* < 0.0001.

Figure 8 shows the displacement values measured for each applied strain on the 5 kPa elastic substrate above and below the collagen layer. As expected, the two-way ANOVA showed a significant increase in the displacement values when increasing the *applied strain* (*p* < 0.0001). However, no significant differences emerged when comparing the displacement values measured above and below the collagen layer.

**Figure 9:**
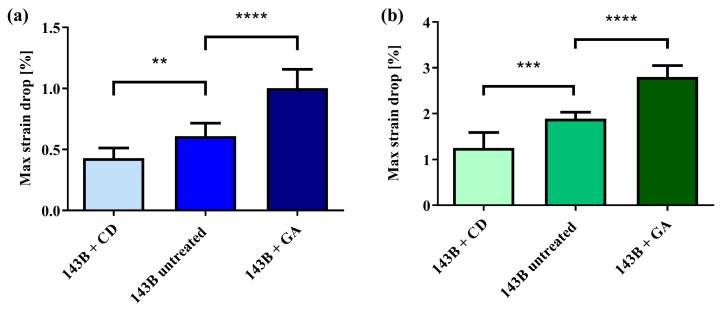
(**a**) Maximum *strain drop* values for treated and untreated 143B cells cultured on the 5 kPa substrate at 1% of applied strain. (**b**) Maximum *strain drop* values for treated and untreated 143B cells cultured on the 5 kPa substrate at 4% of applied strain. Data are reported as mean ± SD. **: *p* < 0.01; ***: *p* < 0.001; ****: *p* < 0.0001. 6 ≤ *n* ≤ 11.

Figure 9 shows the maximum *strain drop* values obtained when testing the untreated 143B cells in comparison to the 143B cells treated with glutaraldehyde or with cytochalasin-D, plated on 5 kPa substrates at 1% and 4% of applied strain.

**Figure 10:**
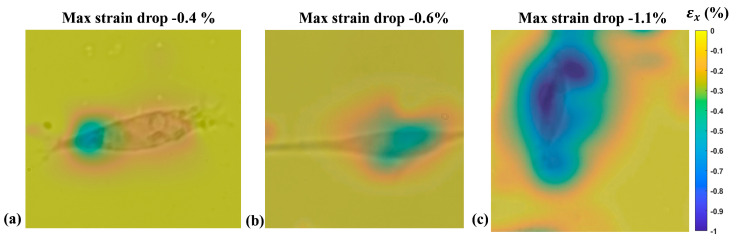
A representative example of the *strain drop* measured for 143B cells adherent on the 5 kPa substrate and subjected to an external strain of 1%. (**a**) 143B treated with cytochalasin-D; (**b**) Untreated 143B; (**c**) 143B treated with glutaraldehyde.

Results showed that, for the two applied strain values, the system was capable of capturing the difference in the cell elasticity induced by the two chemical treatments. Indeed, in both cases, the one-way ANOVA showed a significant difference (*p* < 0.0001) in the maximum *strain drop* between the considered cells. The post hoc tests pointed out a significant reduction (*p* < 0.01 for 1% and *p* < 0.001 for 4%) in the maximum *strain drop* for the cells treated with cytochalasin-D compared to the untreated cells. At the same time, a significant increase (*p* < 0.0001 for both 1% and 4%) in the *strain drop* was measured for the cells treated with glutaraldehyde. Figure 10 shows a representative example of the color map of the deformation field obtained from a single adherent 143B cell for each of the three tested groups cultured on a 5 kPa substrate and subjected to 1% of applied strain.

### 3.4. Strain Drop Measurement

**Figure 11:**
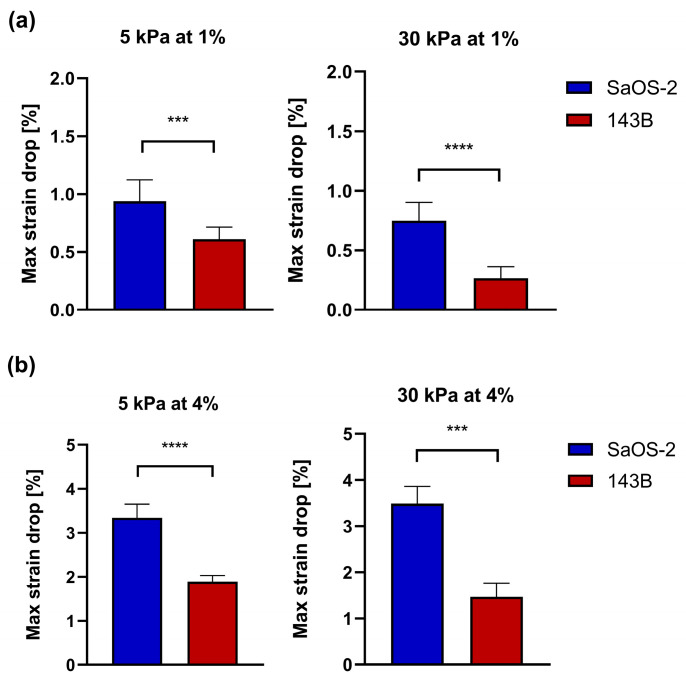
(**a**) Mean ± SD of the *strain drop* for SaOS-2 and 143B cell lines cultured on 5 kPa and 30 kPa substrates deformed at 1%. SD. (**b**) Mean ± SD of the *strain drop* for SaOS-2 and 143B cell lines cultured on 5 kPa and 30 kPa substrates deformed at 4%. 6 ≤ *n* ≤ 21. ***: *p* = 0.0001; ****: *p* < 0.0001.

Having demonstrated the capability of the proposed technique to capture modulations in the stiffness of the adherent cells plated on the same substrate, we tested two OS cell lines with different aggressiveness cultured on two different types of substrates, one resembling the elasticity of the ECM of the lung parenchyma (5 kPa) and one to reproduce the bone mineralized ECM (30 kPa). Figure 11 shows the *strain drop* values measured for SaOS-2 and 143B cells when cultured on 5 kPa and 30 kPa substrates during the application of 1% and 4% of external strain. The results showed an average *strain drop* value lower in 143B cells in comparison with SaOS-2 cells in all the tested conditions. In detail, when the cells were stretched at 1%, the 143B *strain drop* resulted in significantly lower values than that of SaOS-2, at 35% (*p* = 0.0001) and 64% (*p* < 0.0001) on average for the 5 kPa and 30 kPa substrates, respectively (Figure 11a). Similarly, during the application of 4% of external strain, the *strain drop* measured for the 143B cell lines resulted to be, on average, 43% (*p* < 0.0001) and 58% (*p* = 0.0001) lower than those measured for the SaOS-2 cells when cultured on 5 kPa and 30 kPa substrates, respectively (Figure 11b).

## 4. Discussion

The aim of this work was to develop an optical system for measuring the *strain drop*, a parameter linked to cellular elasticity, of two osteosarcoma cell lines when adherent to elastic substrates with different stiffnesses. Cells are highly sensitive to the mechanical properties of the substrate where they adhere [25,28] and, in presence of an external deformation of the substrate, they react with a resistance that appears in terms of a decreased substrate strain beneath the cell body, which is directly correlated to the mechanical properties of the cells, such as the cellular elasticity. Even if the proposed approach does not provide a quantitative description of the cell mechanical properties in absolute terms (e.g., the elastic modulus), it can clearly distinguish the relative differences in cell elasticity. This approach is essential for understanding the pathophysiology of various diseases, for example, in cancer progression and metastasis. Notably, cancer cells often exhibit altered mechanical properties compared to healthy cells [53,54,55].

The proposed system was able to maintain an unchanged cellular morphology and functionality, as no component of the device came into contact with the cells, unlike other invasive methods used [13,14]. Research conducted by Bartalena et al. [15] resulted in the development of another optical method for assessing the *strain drop* of individual adherent cells. This system enables the quantification of the underlying substrate’s extensibility when subjected to deformation, particularly for single cells on a substrate with an elasticity of 5 kPa through a radial stretching. Unlike this complex platform, our device employs a simpler approach involving linear stimulation. In order to take into account the cell–matrix interplay, we also customized the stretching chamber by accommodating elastic substrates with variable elasticities of 5 kPa and 30 kPa, in our case, but potentially any desired physiological value. The characterization of the silicone chamber demonstrated that the silicone substrate strain was consistently homogeneous on the entire base of the chamber, ensuring a uniform transfer of the strain on the adherent cells. Interestingly, the global analysis conducted in our previous work [42], which stands for a study of different zones across the silicon substrate, was confirmed by the local study, which allows for the study of the strain distribution in a dimension close to that of single cells, and the small differences obtained here are probably due to the higher magnification employed, which introduced bigger focus errors. We also demonstrated that the presence of the collagen layer does not significantly alter the deformation experienced by the cells, ensuring that the cells were only sensitive to the stiffness of the silicone chamber.

Generally, the system was shown to be sensitive enough to perceive differences in the cell’s elasticity induced by chemical treatments. This outcome also supports the crucial conditions that cells must adhere on the substrate, and therefore also on the customized ones, to be suitable for *strain drop* measurements. Research conducted by Bartalena et al. [15] demonstrated a similar funding when measuring the *strain drop* values in SaOS-2 cell lines in comparison with the same cells treated with GA and CD when stretched at 8% of external strain, and their results were also confirmed by data obtained with the AFM experiments. Conversely, here, we were able to demonstrate the capability of the system to distinguish differences in the cells’ elasticity, even with very low external strains of 1%.

As concerns the results obtained from the SaOS-2 and 143B cells tested on the 5 kPa and 30 kPa substrates, they point out a lower *strain drop* value (in modulus) for the high-metastatic 143B cells with respect to the low-aggressive SaOS-2 cells. These results are in agreement with a study by K. Sangwoo et al. [8], which demonstrated that different types of cancer cells have a higher Young’s modulus than healthy ones, and that this difference may depend on the histological origin or cancer type. In this context, SaOS-2 and 143B cells have the same histological origin (i.e., the bone tissue) but they can be distinguished based on their aggressiveness. Indeed, SaOS-2 cells are commonly recognized as osteoblast-like cells; therefore, we can speculate that the different *strain drop*s measured here clearly demonstrate the strength of this optical system in distinguishing cancer from normal cells, even from the same tissue of origin.

These results are also consistent with several studies that have deepened several healthy and pathological cell strains [8,56], even if there are also conflicting results which show that cancer cells have greater resistance to applied loads, thereby resulting in being more rigid or even unchanged [9].

In conclusion, the present study allowed for the measurement of a parameter closely related with the elasticity of adherent cells. This permitted us to exploit the adhesion to stretch the cells, thus avoiding any interference from external instrumentation, and to merge this evaluation with the study of the cells’ adhesion on different substrates to recall the ECMs of different tissues/organs. Indeed, the measurement system here proposed might include substrates with any other physiological elasticity, thus allowing us to test cells from any pathological conditions.

## 5. Conclusions

In this article, a system for the measurement of cells’ *strain drop*, a parameter correlated with cell elasticity, was designed, developed, and characterized. Cell elasticity was recently proposed as a potential biomarker for several diseases, and these novel techniques are therefore essential for a deep study of cell mechanics in different pathological conditions. The system here proposed was based on a stretching device and the simultaneous measurement of the cellular ability to resist actively the underlying matrix strain through the use of an imaging system. Of note, this technique was non-invasive, since it took advantage of the adhesion forces already existing between the cell and the underlying substrate. The cell’s adhesion was also exploited to study the interplay between the cells and the substrate, representative of the tissue/organ ECM.

The platform was demonstrated to be able to discern the stiffness of treated and untreated osteosarcoma 143B cells based on resistance to the deformation of a *sufficiently soft*, 5 kPa substrate, especially at a 1% low deformation. To make the experiments as accurate as possible, preliminary tests were performed to validate the capability of the control algorithm in repositioning the chamber in the initial field of view. Other tests have been performed to validate the collagen coating homogeneity and its negligible role on the cells’ strain sensing. The measurement technique was then employed to test two osteosarcoma cell lines, one considered osteoblast-like, the SaOS-2, and one known to metastasize to the lung tissue, the 143B, a tissue with a very different stiffness than the tissue of origin, i.e., bone. Experimental results showed that the two tested cell lines have a different sensitivity to mechanical stimuli, with the 143B cells being intrinsically more elastic than their SaOS-2 counterpart, likely due to their high-aggressive nature. The current study also aimed at proposing a novel approach combining the measurement of mechanical parameters and mechanosensitivity with the interplay between cells and the tissue-specific ECM, an approach that may be applied to several other pathological conditions in which a deregulation of the mechanotransduction or the mechanoenvironment has been described.

## Figures and Tables

**Table 1:** ROIs (8 × 8 nodes) for each of the 5 kPa samples tested when applying 1% and 4% of stretching, *n* = 9.

Applied Strain	Sample	Local Homogeneity Values for the 5 kPa Elastic Substrate		
		Mean (%)	SD (%)	CV (%)
**1%**	1	1.00	0.09	9.22
	2	1.05	0.12	11.05
	3	0.94	0.09	9.25
**4%**	1	3.96	0.31	7.82
	2	3.91	0.45	11.20
	3	3.76	0.32	8.48

**Table 2:** Mean, SD, and CV of the axial strain measured on the 9 ROIs (8 × 8 nodes) for each of the 30 kPa samples tested when applying 1% and 4% of stretching, *n* = 9.

Applied Strain	Sample	Local Homogeneity Values for the 30 kPa Elastic Substrate		
		Mean (%)	SD (%)	CV (%)
**1%**	1	1.02	0.08	7.70
	2	1.04	0.08	8.01
	3	0.99	0.07	7.35
**4%**	1	4.00	0.36	8.84
	2	4.11	0.26	6.42
	3	3.92	0.29	7.45

## Data Availability

The data are available upon request.
